# Food consumption patterns and nutrient adequacy across lifecycle stages in Bangladesh

**DOI:** 10.1371/journal.pone.0359205

**Published:** 2026-09-28

**Authors:** Md. Hafizul Islam, Nazma Shaheen, Masum Ali, Abira Nowar, Saiful Islam, Syed Shahadat Hossain, Md. Ruhul Amin

**Affiliations:** 1 Institute of Nutrition and Food Science, University of Dhaka, Dhaka, Bangladesh; 2 Center for Non-communicable Diseases and Nutrition, BRAC James P Grant School of Public Health, BRAC University, Dhaka, Bangladesh; 3 Nutrition and Health Science, Laney Graduate School, Emory University, Atlanta, United States of America; 4 Institute of Statistical Research and Training, University of Dhaka, Dhaka, Bangladesh; Necmettin Erbakan Üniversitesi: Necmettin Erbakan Universitesi, TÜRKIYE

## Abstract

**Objective:**

The study aimed to estimate energy, macro, and micronutrient intakes and to compute the probability of adequacy (PA) of 11 selected micronutrient intakes by age, sex, and vulnerabilities.

**Methods:**

This study analyzed dietary data from two nationally representative surveys in Bangladesh: the BIHS 2015 (N = 13,086; rural only) and the NSB 2017–2018 (N = 3,116; rural and urban, all divisions). Participants included individuals of all ages and both sexes. Primary outcomes were energy intake and micronutrient adequacy, estimated using usual intake and mean probability of adequacy (MPA) for 11 micronutrients. Secondary outcomes included intake of 13 food groups, macronutrients, dietary fiber, and food-group contributions to nutrient intake, standardized to the individual level using adult male equivalents.

**Results:**

The study showed that cereals dominated diets, with low intake of fruits, leafy vegetables, pulses, milk, and nuts. Nutrient adequacy was low for calcium, riboflavin, vitamin B12, folate, zinc, and vitamin A, except for niacin and vitamin B6. The median MPA across 11 micronutrients was low for all age and physiological groups. Females had lower odds (AOR: 0.58, 95% CI: 0.49, 0.70, p < 0.001) of having higher MPA than males. Adolescents (10–18 years) were less likely (AOR: 0.57, 95% CI: 0.37, 0.88, P = 0.01) to have higher MPA compared to children (1–5 years). Participants from large families (>4 members) had lower odds (AOR: 0.70, 95% CI: 0.57, 0.86, p = 0.001) of having higher MPA than those from smaller families. Children under 2 years were less likely (AOR: 0.51, 95% CI: 0.41, 0.64, p < 0.001) to have higher MPA than women of reproductive age (WRA).

**Conclusion:**

The study found cereal-dominated diets with low consumption of fruits, leafy vegetables, pulses, milk, and nuts, potentially contributing to widespread inadequacy of essential micronutrients across all age groups. Policies and strategies should promote the production, affordability, accessibility, and consumption of nutrient-dense foods, particularly pulses, fruits, nuts and seeds, leafy vegetables, and dairy products, to improve micronutrient adequacy.

## Introduction

Bangladesh has made significant improvements in undernutrition, while the rates of stunting, and underweight and micronutrient inadequacy among children remain prevalent [1]. Similarly, clinical vitamin A deficiency disorder (VAD) is prevalent in pregnant women and women of reproductive age (WRA) [2]. Pregnant and lactating women also have a high prevalence of anemia and subclinical VAD [3]. Certain micronutrient deficiencies, such as vitamin A, folate, iron, calcium, and zinc, are common among women in Bangladesh. These deficiencies are associated with a variety of unfavorable health outcomes, including growth restriction, birth defects, impaired cognitive function, and an increased risk of morbidity and mortality [4,5]. Furthermore, the triple burden of malnutrition (undernutrition, micronutrient deficiencies, and overnutrition) has become increasingly concerning in recent years. Overnutrition and obesity are linked to several nutrition-related non-communicable diseases (NR-NCDs), such as diabetes mellitus, hypertension, cardiovascular diseases, and non-alcoholic fatty liver disease (NAFLD) [6–9]. Dietary patterns characterized by high consumption of energy-dense, nutrient-poor foods contribute directly to this triple burden, exacerbating both undernutrition and NCD risk.

The recommended infant and young child feeding (IYCF) procedures are not followed to the fullest extent. The latest national estimates showed that only dietary diversity and acceptable diet quality of children aged 6–23 months are very low [1]. Different micronutrient deficiencies among women are caused mainly by insufficient intakes of micronutrient-rich foods, which are linked to monotonous, low-quality diets [10,11]. The prevalence of adequate intakes was < 50% for iron, calcium, riboflavin, folate, vitamin B-12, and calcium [10,12]. Another study conducted in Bangladesh among rural WRA showed that the prevalence of inadequate dietary intake of calcium, zinc, and energy was significantly higher in adolescent girls than in adult women [13]. Similar studies conducted in neighboring and low-income countries showed a high prevalence and low adequacy of vitamin A, vitamin E, folate, and vitamin B-12, and calcium in children [14–18].

To combat the existing problems of dietary inadequacies, every food-based dietary guideline, including Bangladesh, recommends consuming an age- and sex-specific balanced and healthy diet. Because human nutrition is so complex, a person’s definition of a “healthy diet” can vary significantly based on their genetic makeup, age, sex, environment, and cultural background [19–21]. Such complexity diverts attention to the need to depend on energy availability and adequacy to address hunger and food insecurity. But when obesity, malnutrition, and food insecurity are putting a triple burden on society, diets must be modified to ensure that everyone gets the energy and nutrients they need. To achieve this, it is necessary to measure the energy and nutritional content of habitual diets, compare them to estimated average requirements (EARs), and advocate for more desirable diets that are both culturally acceptable and reasonably priced for people of all economic levels. Furthermore, it is crucial to find gaps in the nutrition density of people’s diets during various life cycles due to their varying needs at different times of their lives.

A few recent studies in Bangladesh have highlighted trends in nutrient adequacy among rural populations, focusing on different age, sex, and wealth groups [10,11,13,22]. While some vulnerable groups, such as adolescents, pregnant, and lactating women, were included in these analyses [12,23], there remains a lack of comprehensive assessment across all key vulnerable subpopulations, particularly using nationally representative data. Moreover, the socio-demographic determinants of nutrient adequacy were either not the primary focus or were insufficiently explored. To address these gaps, the present study aimed to determine the food consumption patterns and diet quality in terms of the probability of nutrient adequacy for Bangladeshi people of different ages and life stages. It utilized two complementary datasets,the Bangladesh Integrated Household Survey (BIHS) 2015, which is rural representative, and the Nutrition Survey of Bangladesh (NSB), which is nationally representative. This approach enables a broader analysis of food consumption patterns and diet quality, assessed through the probability of nutrient adequacy across different age groups and vulnerable populations. Moreover, the study aimed to explore key socio-demographic determinants influencing nutrient adequacy to support evidence-based policy design and implementation.

## Methodology

### Data sources and subjects

Using data from the Nutrition Survey of Bangladesh (NSB) 2017–18 [24] and Bangladesh Integrated Household Survey (BIHS) 2015 [25], the present study aimed to determine the food consumption patterns and diet quality for Bangladeshi people of different ages and life stages. The datasets were treated as independent and separately analyzed, and no pooled or merged dataset was created. The use of data from two different surveys provides a more comprehensive assessment of food consumption patterns and diet quality across different age groups and life stages. While NSB 2017–18 offers nationally representative dietary intake data with detailed nutrient consumption, BIHS 2015 provides in-depth household-level food acquisition and consumption data with socioeconomic linkages. Using both datasets allows for cross-validation of findings and helps bridge the gap between individual dietary intake and household food availability. This combined approach strengthens the reliability of our analysis and provides a more detailed perspective on nutrient adequacy across diverse population groups. Key features of the two surveys are presented in supplementary Table 1 in S1 File, highlighing their differences in survey design, dietary assessment methods, sample characteristics, and limitations.

**Nutrition Survey of Bangladesh (NSB) 2017−18**: The Institute of Nutrition and Food Science, University of Dhaka, conducted Nutrition Survey of Bangladesh (NSB) 2017−18. All surviving men and women from the previous surveys (81–82; 2001–02; 2007–2008) were included in the NSB 2017–18 survey, regardless of where they lived in Bangladesh. To improve population representativeness and concentrate more attention on urban residents, an extra sample of 30 villages X 30 households from among the 699 2007/08 villages was also included in the survey. These villages are statistically chosen using the Probability Proportion to Size (PPS) approach from the places visited in 2007–08. The food consumption of 3541 people was included in this study. The NSB survey collected individual-level intake of the household members using the weighed food record method.

**Bangladesh Integrated Household Survey (BIHS)-2015 Survey**: The International Food Policy Research Institute (IFPRI) of Bangladesh carried out the Bangladesh Integrated Household Survey (BIHS). This nationally representative panel survey produced three rounds of panel datasets. Three rounds were conducted: the first in 2012, the second in 2015, and the third in 2018. Within the scope of the Integrated Multipurpose Sample (IMPS) design, this survey employed a two-stage stratified random sampling technique. It comprises individual-level 24-hour recall dietary estimates from the same individuals and a household survey. A total of 5,503 households in this study were representative of rural Bangladesh. We used the BIHS 2015 and NSB 2017–18 datasets to ensure temporal alignment and access to detailed dietary data required for estimating the probability of nutrient adequacy. BIHS 2015 includes two non-consecutive 24-hour dietary recalls, enabling robust estimation of usual intake. Within one or two weeks, about 10% of the households were revisited to collect additional dietary data.

### Dietary data collection and management procedures

Using the food weighed record system, the NSB 2017−18 survey gathered the individual consumption of each household member. The cooked food item was converted to the raw ingredients of the consumed food item using a conversion factor. The amount of uncooked food that the person consumed was measured using kitchen scales and household utensils. We used the modules on intra-household food consumption (including quantitative data on portions of cooked composite meals consumed by individuals within the household) and household food consumption (including quantitative data on raw ingredients used to prepare composite meals) for the BIHS survey. Data on food intake and intra-household food were collected from the individual primarily in charge of meal preparation using a 24-hour recall technique. The intra-household data set only provided the cooked weight of composite foods (menu items) consumed by the individual (e.g., fish and vegetable curry), so the following computation was required to determine the equivalent amount of raw ingredients consumed by each individual to estimate nutrient intakes using food composition data.


$$\begin{array}{c} \;Weight\;of\;raw\;{ingredient}_{\left(individual\right)}\;=\;weight\;of\;raw\;{ingredient}_{\left(household\right)}\times \frac{{Cooked\;weight\;of\;composite\;food}_{\left(individual\right)}\;}{{Cooked\;weight\;of\;composite\;food}_{\left(household\right)}} \end{array}$$

### Data processing and calculation of nutrient intake

Using several procedures, dietary data and background information were harmonized to create analytical data sets. The main procedures were as follows: a) retrieving the dataset, which involved finding and obtaining pertinent socio-demographic, socioeconomic, and dietary variables from the NSB and BIHS datasets; b) matching food items to determine the relative contributions of different food items and food groups to the nutrient intakes of households, the reported food items were matched with corresponding food items in the Food Composition Table for Bangladesh (FCTB-2013). Foods from the regional FCTs were matched with individual foods that were not identified in the FCTB. This method used the FCTB to match 81% of the food, while the USDA was used for the remaining 19% of matches.; c) The FCTB was used to classify different food items to food groups (e.g., fruits, vegetables); d) The adult male equivalent (AME) was used to individualize household food and nutrient consumption from the BIHS dataset based on age, and sex of household members. This approach allows for a more accurate estimation of per capita food intake, assuming equal distribution among all household members. The datasets were cleaned, processed, shaped, and combined to create the final analytical dataset. This study employed the same AME as previous research conducted in Bangladesh [26]. While this approach improves upon equal‑share allocation, we acknowledge that it cannot capture gender‑, age‑, or role‑based disparities in intra‑household food distribution. Estimates for women, children, and vulnerable groups may therefore be affected. The data were processed and managed using software such as STATA and SPSS. STATA was used to calculate the probability of nutrient adequacy, while SPSS was employed for the statistical analyses.

### Dietary variables

Dietary indicators that recorded a person’s intake were included. Considering the importance to public health, we incorporated 13 food groups, 4 macronutrients, 11 micronutrients, and total energy as dietary parameters. Cereals and their products; legumes, pulses, and their products; vegetables and their products, leafy and non-leafy; starchy roots, tubers, and their products; nuts, seeds, and their products; spices, condiments, and herbs; fruits; fish, shellfish, and their products; meat, poultry, and their products; eggs and their products; milk and its products; and fats and oils are among the 13 food groups. Protein, fat, carbohydrates, and dietary fiber are the four macronutrients. Calcium, iron, zinc, thiamine, riboflavin, niacin EQ, vitamin B6, folate, vitamin B12, L-ascorbic acid, and vitamin A (RAE) are among the 11 micronutrients.

### Estimation of the probability of adequacy of nutrients

The National Institute of Nutrition, India’s estimated average requirements (EARs) were used to assess the probability of adequacy (PA) of the 11 micronutrients [27]. These ICMR‑NIN Indian EAR values were chosen because Bangladesh does not yet have its own nationally endorsed nutrient requirement standards, and the Indian EARs are regionally relevant, reflecting similar dietary patterns, food systems, and environmental exposures. Moreover, the food-based dietary guidelines of Bangladesh also report these ICMR‑NIN Indian EAR values. We determined the average intake to assess each person’s adequacy of dietary intake. The usual intake was estimated by adjusting for intra- and inter-individual variation using log-transformed intake data and ANOVA-based variance decomposition, followed by back-transformation to derive habitual intake distributions; repeated dietary measurements were available BIHS 2015, which allowed for correction of within-person variation and improved estimation of nutrient adequacy, as unadjusted intake data can overestimate the prevalence of inadequacy due to day-to-day variability [28]. The RDA and EAR values were used to compute the standard deviation (SD) (RDA-EAR/1.96). The mean probability of adequacy (MPA) for 11 micronutrients was determined by averaging the micronutrients’ adequacy. Nutrient adequacy for women who were pregnant or lactating was determined separately.

### Statistical analysis

The BIHS complex survey design has been utilized to compute the weighted estimation. According to the Goldberg criteria for age and sex, lower and higher intake were found and counted as a missing value in the present study. To improve data quality, implausible energy intake values were excluded using commonly applied plausibility thresholds for adult populations. Specifically, individuals with reported daily energy intake below 2,092 kJ (<500 kcal/day) or above 20,920 kJ (>5,000 kcal/day) were considered outliers and excluded from the analysis. These thresholds were applied uniformly across all adult age groups included in the study and were not applied to children or adolescents. The exclusion of extreme values resulted in the removal of a small number of observations and did not materially alter the overall sample size or the interpretation of subgroup comparisons. The conventional procedure was used to clean the individual intake with lower and higher intake. We estimated and compared the average dietary consumption by demographic strata, such as age, sex, region, etc. We have utilized log transformation for the non-normal data to get the usual intake. We performed a back transformation after modifying the inter and intra variation using the log distribution to determine an individual’s typical intake. Because most of the data were skewed, comparisons for the consumption of food groups and nutrients between age groups and genders were made using the Kruskal-Wallis test, and the data were reported as medians and interquartile ranges (IQR). The study employed multivariate logistic regression analysis to determine the parameters linked to nutrient adequacy (MPA > 0.5). This cut‑point is an operational choice supported by prior literature, and alternative thresholds could be explored in future research. The variable selection was guided by the threshold p < 0.2 in the univariate analysis. Multicollinearity among the independent variables was assessed, and a variance inflation factor (VIF) of less than 10 was considered to indicate the absence of multicollinearity. The level of significance was considered at p < 0.05. Data analyses were performed using the software package SPSS (version 25.0, SPSS Inc., Chicago, IL, USA).

### Ethical considerations

This study used publicly available, de-identified secondary data. No primary data were collected and no identifiable information was accessed. The original surveys obtained ethical approval from relevant institutional review boards and informed consent from participants. Thus, this secondary analysis of anonymized publicly available data did not require additional ethical approval.

## Results

### Socio-demographic characteristics of study participants

The sociodemographic characteristics of study participants from the NSB 2017−18 survey and the BIHS 2015 are presented in Table 1. According to the NSB 2017−18 survey, the majority (55%) of the participants were between 19−60 years, followed by 1−5 years (17.8%) and 10−18 years (14.3%). The proportion of male and female participants was almost equal. Most of the participants (65%) were from a small family (≤4 members). One-third had no formal education, and a similar proportion had primary education. On the other hand, in the BIHS 2015, more than half (54.6%) of the participants were women of reproductive age, and about one-fourth (24.8%) were in pre-adolescent periods. Most participants (82.7%) were female in the BIHS 2015, and more than half (52.7%) were from small families. 36% of them had a primary level of education, and about one-third (34.4%) had a secondary level. According to the NSB 2017−18 survey, 9% were self-employed, 6.4% were involved in trade, and 6% were in the farming sector. On the contrary, 17.1% of the participants of the BIHS 2015 survey were involved in farming.

**Table 1:** Socio-demographic characteristics of study participants from the NSB 2017−18 survey and the BIHS 2015 survey.

Variables	NSB 2017−18 survey (N = 3116)		BIHS 2015 survey (N = 13086)	
Age (years)	n	%	n	%
1-5	555	17.8	–	–
6-9	285	9.1	–	–
10-18	446	14.3	–	–
19-60	1714	55	–	–
60+	116	3.7	–	–
**Vulnerable groups**				
Children under 2	–	–	1271	9.7
Pre-adolescents (10–14)	–	–	3246	24.8
Women of Reproductive Age (15–49 years)	–	–	7146	54.6
Pregnant	–	–	283	2.2
Lactating	–	–	1140	8.7
**Gender**				
Male	1559	50	2266	17.3
Female	1557	50	10820	82.7
**Family size**				
Less or equal to 4	2024	65	6184	52.7
More than 4	1092	35	5561	47.3
**Literacy**				
No formal education	1050	33.7	3422	26.2
Primary level (grade 1–5)	1069	34.3	4714	36
Secondary level (grade 6–10)	780	25	4504	34.4
Higher secondary level (grade 11–12)	113	3.6	359	2.7
Graduate	104	3.3	87	0.7
**Occupation**				
Daily wage labor	97	3.1	273	2.1
Monthly salaried work	73	2.3	235	1.8
Self-employed	288	9.2	148	1.1
Trader (small store, roadside stall, large shop, fish trader)	199	6.4	55	0.4
Production (food processing, handicrafts, small industry)	3	0.1	83	0.6
Farming	186	6	2235	17.1
Non-earning activities (children, students, housewife, physically/mentally challenged)	2270	72.8	10057	76.9

NSB = Nutrition Survey of Bangladesh; BIHS = Bangladesh Integrated Household Survey.

### Food consumption and nutrient intake

Table 2 shows the intake of different food groups and macro and micronutrients of Bangladeshi people according to the NSB 2017–18 survey. Intake of various food groups increased with age, and the highest intake level was observed in the 19–60 age group. However, the median intake of different food groups was lower in children 6–9 years old than children 1–5. After age 60, the intake level of various food groups decreased again. Cereal dominated the diet of Bangladeshi people, and the average cereal intake was 435 g, 365 g, and 332 g among 19–60-year-olds, 60 + year-olds, and 10–18-year-olds participants, respectively. Intake of animal foods was around 94 g among 19–60-year-old adults and 69 g for 10–18 age groups. Consumption of vegetables, fruits, fats and oils, pulses, and other remaining food groups was also highest among adults (19–60 years) and lowest among under-5 children. Similarly, in the case of dietary energy, macro, and micronutrient intake, an increasing trend was found with the increase of age. The median intake of energy was 2119 kcal among the adults (19–60 years), 1846 kcal among the elderly (60 + years), and 1599 among 10–18 years.

**Table 2:** Median (IQR) daily food groups and nutrient intake by different age groups according to NSB 2017−18 survey.

	1-5 years	6-9 years	10-18 years	19-60 years	60 + years
Food Groups (g/day)	Median (IQR)	Median (IQR)	Median (IQR)	Median (IQR)	Median (IQR)
Cereals	135.7 (89.2, 188.8)	231.3 (182.2, 282.7)	331.9 (274.8, 398.4)	435.5 (372.6 501.3)	364.8 (290.1, 457.9)
Pulse^†^	4.81 (0, 152.89)	7.10 (0, 98.34)	12.12 (0, 155.04)	16.09 (0, 313.93)	18.16 (0, 315)
Non-leafy vegetables	63.4 (26.0, 154.3)	48.1 (22.2, 83.8)	67.8 (32.8, 127.5)	101.4 (50.2, 170.9)	63.4 (26.0, 154.3)
Leafy vegetables^†^	6.02 (0, 430.82)	7.51 (0, 185.03)	13.24 (0, 250.3)	21.26 (0, 1009)	35.85 (0, 530.51)
Starchy roots	40.4 (0, 95.9)	27.7 (7.9, 58.5)	47.3 (0, 90.1)	62.8 (12.8, 111.9)	40.4 (0, 95.9)
Nuts and seeds^†^	0.07 (0, 13.18)	0.25 (0, 21)	0.89 (0, 82.62)	0.83 (0, 100.14)	9.24 (0, 279.47)
Spices	4.9 (2.9, 8.0)	2.30 (1.2, 4.2)	3.8 (1.9, 7.0)	4.6 (2.5, 7.9)	4.9 (2.9, 8.0)
Fruits^†^	10.29 (0, 284)	7.72 (0, 327)	8.27 (0, 365)	9.83 (0, 701)	8.82 (0, 180)
Fishes	33.2 (0, 72.6)	28.2 (2.2, 50.1)	37.0 (0, 67.9)	52.5 (0, 91.7)	33.2 (0, 72.6)
Meats^†^	9.81 (0, 135.64)	13.91 (0, 219.55)	20.20 (0, 273.92)	27.68 (0, 339.49)	18.41 (0, 212.7)
Eggs^†^	9.79 (0, 165)	6.41 (0, 80.48)	10.51 (0, 112.39)	11.50 (0, 213.86)	5.45 (0, 112)
Milk^†^	32.28 (0, 531)	8.64 (0, 321.15)	8.90 (0, 342.03)	10.46 (0, 418)	7.56 (0, 170.7)
ASF	58.8 (21.5, 118.1)	50.1 (25.7, 79.6)	69.3 (35.2, 116.1)	93.7 (54.1, 150.9)	58.8 (21.5, 118.1)
Fats and oils	23.9 (13.5, 35.0)	13.9 (9.1, 20.8)	19.1 (12.9, 31.1)	29.1 (19.5, 43.2)	23.9 (13.5, 35.0)
**Nutrients**	**Median (IQR)**	**Median (IQR)**	**Median (IQR)**	**Median (IQR)**	**Median (IQR)**
Energy (Kcal)	699.2 (494.9, 937.6)	1104.3 (862.1, 1374.2)	1599.0 (1286.1, 1886.6)	2118.8 (1804.8, 2467.2)	1845.8 (1445.0, 2168.1)
Protein (g)	19.3 (13.1, 26.7)	29.0 (21.5, 38.7)	41.7 (32.5, 53.0)	55.5 (45.1, 68.7)	46.7 (34.8, 58.5)
Total fat (g)	13.5 (7.8, 20.5)	19 (13.4, 30.0)	28.1 (18.2, 39.6)	38.7 (26.6, 55.5)	30.2 (20.8, 43.2)
Carbohydrate (g)	118.6 (82.2, 161.6)	194.3 (150.3, 237.9)	277.5 (225.3, 324.8)	364.3 (316.0, 424.6)	321.6 (251.0, 391.1)
Total dietary fiber (g)	6.9 (4.6, 9.6)	11.6 (9.1, 14.7)	17.1 (14.1, 21.6)	23.0 (19.1, 27.8)	20.8 (15.8, 25.0)
Calcium (mg)	73.5 (39.9, 167.9)	94.8 (59.7, 165.9)	155.8 (99.9, 290.4)	191.5 (126.7, 357.9)	144.3 (97.4, 315.8)
Iron (mg)	2.6 (1.7, 3.8)	4.0 (3.1, 5.6)	6.6 (4.8, 8.9)	8.4 (6.5, 11.2)	7.8 (5.8, 10.5)
Zinc (mg)	3.2 (2.2, 4.3)	4.8 (3.7, 6.1)	7.1 (5.7, 8.6)	9.3 (7.7, 11.1)	7.9 (6.1, 9.8)
Vitamin A, RAE (mcg)	38.9 (9.2, 103.4)	34.3 (14.5, 84.3)	72.5 (28.6, 160.6)	81.5 (35.3, 183.0)	67.2 (26.7, 222.1)
Vitamin E (mg)	1.9 (1.1, 3.1)	3.2 (2.3, 4.9)	4.7 (3.2, 7.1)	6.6 (4.6, 9.4)	5.4 (3.5, 8.1)
Thiamin (mg)	0.4 (0.3, 0.6)	0.7 (0.5, 0.8)	1.0 (0.8, 1.2)	1.3 (1.1, 1.6)	1.0 (0.8, 1.5)
Riboflavin (mg)	0.2 (0.1, 0.4)	0.3 (0.2, 0.4)	0.5 (0.3, 0.6)	0.6 (0.5, 0.8)	0.5 (0.4, 0.7)
Niacin quivalent (mg)	8.9 (5.9, 12.3)	14.1 (10.7, 18.2)	20.5(16.0, 25.2)	27.2 (22.6, 33.3)	22.5 (16.0, 29.4)
Vitamin B6 (mg)	0.5 (0.3, 0.7)	0.8 (0.6, 1.0)	1.1 (0.9, 1.4)	1.5 (1.2, 1.8)	1.3 (0.9, 1.6)
Folate (mcg)	50.7 (33.3, 86.1)	80.6 (53.9, 157.4)	112.1 (78.4, 194.3)	147.8 (106.6, 254.3)	138.8 (92.3, 610.4)
L-ascobic acid (mg)	9.3 (4.9, 17.1)	17.6 (9.9, 30.2)	27.8 (14.9, 50.1)	37.3 (22.0, 68.2)	37.8 (13.8, 70.1)
Vitamin B12 (mcg)	0.4 (0, 1.0)	0.4 (0, 1.0)	0.7 (0, 1.7)	0.8 (0, 1.9)	0.4 (0, 1.2)

†The mean (min, max) is reported as the median value is zero (0).; IQR: Interquartile range, RAE = Retinol activity equivalent.

The median daily food groups and nutrient intake by different vulnerable groups according to the BIHS 2015 survey are presented in Table 3. Again, cereal intake dominated the diet of all vulnerable groups. Naturally, children had the lowest intake among all the vulnerable groups. A higher intake of cereals, starchy roots, fish, fats, and oils was observed among lactating mothers compared to women of reproductive age and pregnant mothers. However, the intake of non-leafy vegetables and animal-source foods (especially fish) was highest among pregnant women. An increasing trend was found in dietary energy, macro, and micronutrient intake with the participants’ age increase. The highest median intake of protein, fat, and L-ascorbic acid was observed among pregnant women. In contrast, the median energy intake, carbohydrate, and calcium were highest among lactating mothers. On the other hand, a lower intake of energy, macro, and micronutrients was noted among the WRA compared to the pregnant and lactating women.

**Table 3:** Median (IQR) daily food groups and nutrient intakes by vulnerable groups according to the BIHS 2015 survey.

	Children under 2 years	Pre-adolescents (10–14 years)	WRA (15–49 years)	Pregnant women	Lactating women
Food Groups (g/day)	Median (IQR)	Median (IQR)	Median (IQR)	Median (IQR)	Median (IQR)
Cereals	79.1 (34.9, 129.7)	380.4 (304.2, 472.0)	463.3 (380.0, 555.7)	450.9 (362.5, 542.2)	502.1 (416.6, 590.5)
Pulse^†^	3.62 (0, 215.24)	12.94 (0, 342.68)	15.45 (0, 404.41)	15.95 (0, 212.42)	15.72 (0, 297.81)
Non-leafy vegetables	11.5 (1.3, 35.1)	104.9 (51.1, 180.8)	137.5 (67.6, 221.0)	150.7 (63.8, 230.6)	143.6 (70.9, 219.2)
Leafy vegetables^†^	5.19 (0, 294.62)	24.80 (0, 747.97)	30.37 (0, 650)	35.09 (0, 650)	32.31 (0, 461.56)
Starchy roots	5.4 (0, 26.6)	70.9 (28.2, 127.8)	81.5 (32.0, 145.8)	79.0 (23.0, 143.3)	93.0 (36.1, 161.9)
Nuts and seeds^†^	0.32 (0, 44.43)	1.78 (0, 303.87)	4.84 (0, 343.93)	3.67 (0, 154.3)	3.81 (0, 343.93)
Spices	0.4 (0, 1.3)	3.1 (1.7, 5.4)	4.0 (2.2, 6.9)	4.7 (2.5, 7.5)	4.1 (2.2, 7.1)
Fruits^†^	17.23 (0, 679.52)	23.51 (0, 936)	23.29 (0, 1095.16)	30.48 (0, 1095.16)	21.0 (0, 1001.98)
Fish	0 (0, 6.9)	15.3 (0, 43.5)	20.4 (0, 52.5)	21.0 (0, 56.0)	21.5 (0, 56.4)
Meat^†^	2.61 (0, 160.67)	9.65 (0, 1484.82)	13.2 (0, 1031.34)	16.11 (0, 558.14)	11.67 (0, 612.39)
Eggs^†^	6.90 (0, 272.25)	7.60 (0, 204.52)	7.54 (0, 398.52)	10.36 (0, 149.17)	7.82 (0, 133.09)
Milk^†^	58.31 (0, 900)	24.79 (0, 558.82)	24.08 (0, 702.86)	40.61 (0, 500)	24.21 (0, 700)
ASF	18.2 (0, 91.3)	43.4 (9.6, 96.2)	50.8 (14.4, 109.6)	61.7 (16.2, 132.7)	52.8 (12.8, 113.4)
Fats and oils	2.9 (0.4, 7.2)	15.9 (9.7, 24.4)	20.5 (12.9, 31.3)	22.6 (13.4, 31.0)	21.1 (13.2, 31.2)
**Nutrients**	**Median (IQR)**	**Median (IQR)**	**Median (IQR)**	**Median (IQR)**	**Median (IQR)**
Energy (Kcal)	584.5 (379.5, 804.8)	1797.8 (1465.0, 2182.0)	2154.7 (1786.1, 2553.2)	2638.4 (2132.7, 2978.7)	2668.2 (2256.7, 3087.6)
Protein (g)	15.2 (10.0, 21.5)	45.4 (36.0, 56.6)	54.1 (43.4, 66.5)	66.2 (51.8, 76.8)	65.3 (55.4, 77.0)
Total fat (g)	11.5 (6.6, 19.4)	26.1 (17.8, 37.9)	31.0 (21.2, 44.8)	38.0 (29.3, 53.4)	35.7 (24.5, 49.6)
Carbohydrate (g)	97.5 (60.8, 133.0)	328.0 (264.7, 400.1)	390.4 (321.5, 468.3)	464.1 (395.9, 556.1)	489.2 (407.2, 563.8)
Calcium (mg)	95.7 (41.9, 209.8)	233.2 (131.5, 406.4)	279.6 (161.4, 485.3)	339.9 (206.2, 525.8)	341.1 (207.5, 569.2)
Iron (mg)	2.7 (1.5, 4.1)	8.7 (6.6, 11.6)	10.6 (8.1, 14.2)	13.0 (10.1, 15.8)	13.0 (10.3, 16.9)
Zinc (mg)	2.4 (1.4, 3.4)	7.8 (6.3, 9.6)	9.5 (7.7, 11.5)	11.7 (9.4, 13.6)	11.8 (9.7, 13.7)
Vitamin A, RAE (mcg)	37.0 (8.8, 110.6)	83.6 (29.6, 245.6)	116.8 (45.1, 316.2)	140.3 (57.9, 391.5)	149.2 (56.0, 357.2)
Vitamin E (mg)	1.2 (0.6, 2.2)	4.0 (2.5, 6.1)	5.0 (3.1, 7.5)	6.6 (3.9, 9.9)	5.6 (3.7, 8.1)
Thiamin (mg)	0.2 (0.1, 0.4)	0.8 (0.6, 1.0)	0.9 (0.7, 1.1)	1.1 (0.8, 1.4)	1.1 (0.8, 1.4)
Riboflavin (mg)	0.2 (0.1, 0.5)	0.6 (0.4, 0.8)	0.7 (0.5, 0.9)	0.7 (0.6, 1.1)	0.8 (0.6, 1.1)
Niacin equivalent (mg)	4.4 (2.7, 6.4)	14.2 (11.2, 17.7)	17.2 (13.8, 20.9)	20.7 (17.7, 25.2)	21.1 (17.6, 24.7)
Vitamin B6 (mg)	0.4 (0.2, 0.6)	1.4 (0.9, 2.0)	1.7 (1.1, 2.3)	1.9 (1.4, 2.5)	2.1 (1.4, 2.9)
Folate (mcg)	50.7 (27.7, 88.9)	161.6 (107.0, 257.0)	190.4 (130.5, 305.2)	226.8 (168.2, 361.5)	237.2 (160.2, 369.4)
L-ascobic acid (mg)	12.0 (5.1, 26.2)	51.7 (30.6, 85.2)	61.2 (36.1, 99.6)	80.2 (50.8, 123.2)	70.9 (46.4, 119.0)
Vitamin B12 (mcg)	0.3 (0, 1.0)	0.4 (0, 1.6)	0.5 (0, 1.9)	0.9 (0, 1.9)	0.5 (0, 2.2)

†The mean (min, max) is reported as the median value is zero (0).; IQR: Interquartile range; WRA: women of reproductive age.

### Contributions of food groups to selected macro and micronutrients

The proportion of different food groups to the daily consumption of specific macronutrients (energy, carbohydrate, protein, and fat) in NSB 2017–18 is presented in Supplementary Figure 1 in S1 File. Cereals are the dominant energy source, contributing 70% of daily caloric intake, followed by edible oils at 14%. Cereals account for 53% of protein intake, followed by fish (18%), meat and eggs (11%), while cereals dominate carbohydrate intake at 90%. Fat intake is mainly provided by edible oils, which account for 74% of total fat consumption, followed by fish (8%).

The contributions of various food groups to the daily intake of particular micronutrients in NSB 2017–18 are shown in Supplementary Figure 2 in S1 File. Cereals are the predominant source of iron (44%) and folate (32%), while they also provide a moderate amount of calcium (22%). Vegetables, including leafy vegetables, supply significant amounts of vitamin A (RAE) (16%) and calcium (15%), with leafy vegetables contributing to vitamin A (RAE) (12%) and iron (5%). Calcium is found in milk products and fish, which provide 21% and 12% of the mineral, respectively. Meat and eggs are the primary source of RAE (24%), with a more negligible contribution to iron (5%). While edible oils and sugars do not contribute to these micronutrients, fruits contribute minimally across all population categories.

### Probability of nutrient adequacy

The median PA was lowest for riboflavin, folate, vitamin B_12_, vitamin C, vitamin A (RAE), and calcium in all age groups, according to the NSB 2017–18 survey (Table 4). However, the highest median PA was observed for niacin, and vitamin B6. The median PA for zinc was only 1%, and iron was only 13%. The median pooled MPA of 11 micronutrients was 20% among the participants. The MPA was significantly higher among the adult participants (19–60 years) compared to the children (1–5 years) and adolescents (10–18 years).

**Table 4:** Probability of adequacy of nutrient intakes (based on EAR) in different ages and vulnerable groups.

Nutrients	NSB 2017−18 survey (Age groups)						BIHS 2015 survey (Vulnerable Groups)					
	1-5 years	6-9 years	10-18 years	19-60 years	60 + years	Pooled	Children (<2 years)	Pre-adolescents (10–14 years)	WRA (15–49 years)	Pregnant	Lactating	Pooled
	Median PA, %						Median PA, %					
Thiamin	3^†a^	6^†b^	5^†b^	15^†b^	15^†a^	2	0^a^	0^b^	3^†c^	1^†bcd^	1^†d^	2^†^
Riboflavin	1^†a^	0^b^	0^b^	0^b^	0^b^	2^†^	2^†a^	0^a^	0^b^	0^ab^	0^a^	0
Niacin	100^a^	100^b^	100^b^	100^b^	100^c^	100	17^†a^	81^b^	100^c^	100^bc^	89^b^	98
Vitamin B6	0^a^	5^†ab^	4^†c^	34^d^	2^†e^	4	7^†a^	14^b^	79^c^	49^bcd^	84^d^	44
Folate	13^†a^	24^†a^	18^a^	9^†a^	6^†a^	2	8^†a^	11^b^	89^c^	5^†abc^	1^b^	43
Vitamin B12	0^†a^	0^†ac^	1^ad^	1^bcd^	0^†a^	1^†^	10^†a^	4^†b^	10^†cd^	7^†abd^	2^†bc^	8^†^
Vitamin C	1^†a^	6^†a^	4^†b^	12^†bc^	13^†ac^	9^†^	14^†a^	60^a^	98^b^	18^ab^	11^†b^	45
Vitamin A	8^a^	7^b^	8^b^	9^bc^	8^d^	8	8^a^	9^a^	11^a^	13^a^	0^a^	10
Iron	1^†ab^	1^b^	3^ab^	22^c^	19^c^	13	2^†a^	9^b^	27^c^	1^†d^	8^d^	19
Zinc	53^ab^	63^ad^	12^†c^	7^†b^	1^†cd^	1	1^a^	22^b^	8^c^	2 cd	5^d^	8
Calcium	0^a^	0^c^	0^ac^	0^b^	0^ab^	0	0^a^	0^a^	0^bc^	0^b^	0^c^	0
MPA	18^a^	19^a^	12^a^	23^b^	19^b^	20	13^a^	28^b^	36^c^	20^be^	21^e^	30

†The mean value is reported as the median value is zero (0).; PA = Probability of adequacy; MPA = Mean probability of adequacy; WRA = Women of reproductive age; Superscript letters indicate results of pairwise comparisons following the Kruskal–Wallis test. Groups sharing the same letter (e.g., aa or bb) are not significantly different (p > 0.05), whereas groups with different letters (ab) differ significantly (p < 0.05).

Similarly, in BIHS 2015, the lowest PA was found for riboflavin, folate, vitamin B_12_, vitamin A (RAE), and vitamin C among all the vulnerable groups (Table 4). Moreover, the PA of iron, calcium, zinc, and thiamine was also low. However, the PA was higher for niacin and vitamin B6 in all the vulnerable groups. Children under two years of age had lower PA for almost all the micronutrients compared to other groups. The pregnant and lactating mothers had the highest PA for iron and zinc compared to others. The median MPA of 11 micronutrients was 30% only. Children under 2 years of age had the lowest MPA than other groups. Although no significant difference was observed in MPA between pregnant and lactating mothers, the women of reproductive age had significantly lower MPA than the former two.

### Socio-demographic predictors of the mean probability of adequacy of different micronutrients

The socio-demographic predictors of the MPA of nutrients obtained from the NSB 2017–18 survey and the BIHS 2015 survey are presented in Table 5. In the NSB 2017–18 survey, the sex, age, household size, residence, and expenditure quintiles of the participants were significantly associated with MPA. The females had lower odds (AOR: 0.58, 95% CI: 0.49, 0.70, p < 0.001) of having higher MPA than males. The adolescents (10–18 years) were less likely (AOR: 0.57, 95% CI: 0.37, 0.88, P = 0.01) to have higher MPA compared to the children (1–5 years). The participants from a large family (>4 members) had less chance (AOR: 0.70, 95% CI: 0.57, 0.86, p = 0.001) of having higher MPA than the participants from a small family. Similarly, the urban residents were found to have lower odds (AOR: 0.79, 95% CI: 0.65, 0.97, 0.023) of having higher MPA compared to the rural residents. Moreover, the second and the middle expenditure quintile participants were less likely to have higher MPA than the lowest expenditure quintile group.

**Table 5:** Socio-demographic predictors of the probability of nutrient adequacy.

Variables	NSB 2017−18 survey		BIHS 2015 survey	
	AOR (95% CI)	P-value	AOR (95% CI)	P-value
**Sex**				
Male	1		–	–
Female	0.58 (0.49, 0.70)	<0.001		
**Age (years)**				
1-5	1			
6-9	0.67 (0.41, 1.07)	0.095		
10-18	0.57 (0.37, 0.88)	0.01	–	–
19-60	0.95 (0.70, 1.30)	0.761		
Above 60	0.96 (0.58, 1.61)	0.884		
**Household size**				
≤4 members	1		1	
>4 members	0.70 (0.57, 0.86)	0.001	1.00 (0.91, 1.09)	0.931
**Education**				
No formal education	1		1	
Primary	1.08 (0.81, 1.44)	0.613	0.98 (0.85, 1.12)	0.73
Secondary	1.33 (0.99, 1.77)	0.057	0.97 (0.85, 1.12)	0.689
Higher secondary and above	1.40 (0.95, 2.08)	0.091	1.14 (0.88, 1.46)	0.317
**Residence**				
Rural	1		–	–
Urban	0.79 (0.65, 0.97)	0.023		
**Expenditure quintiles**				
Lowest	1		1	
Second	0.75 (0.57, 0.99)	0.045	1.41 (1.22, 1.64)	<0.001
Middle	0.62 (0.46, 0.82)	0.001	1.69 (1.45, 1.96)	<0.001
Fourth	0.78 (0.59, 1.02)	0.069	1.74 (1.50, 2.03)	<0.001
Highest	0.87 (0.66, 1.14)	0.307	2.63 (2.26, 3.06)	<0.001
**Vulnerable groups**				
Children under 2 years			0.51 (0.41, 0.64)	<0.001
Pre-adolescents			1.20 (1.07, 1.34)	0.002
WRA	–	–	1	
Pregnant			1.18 (0.68, 2.03)	0.554
Lactating			1.78 (1.40, 2.25)	<0.001

AOR: Adjusted odds ratio; CI: Confidence intervals; WRA: Women of reproductive age.

The BIHS 2015 survey found an increasing trend of MPA with higher expenditure quintiles (Table 5). Children under 2 years of age were less likely (AOR: 0.51, 95% CI: 0.41, 0.64, p < 0.001) to have higher MPA than the WRA. On the other hand, the pre-adolescents (AOR: 1.20, 95% CI: 1.07, 1.34, p = 0.002) and lactating women (AOR: 1.78, 95% CI: 1.40, 2.25, p < 0.001) were more likely to have higher MPA compared to the WRA.

## Discussion

The present study highlighted food groups and nutrient intakes among different age, sex, and vulnerability groups using NSB 2017−18 and BIHS 2015 survey data. The study also presents the probability of micronutrient adequacy and its sociodemographic determinants. The findings highlighted that cereals dominate diets, with low intake of fruits, leafy vegetables, pulses, milk, and nuts. Nutrient adequacy was low for calcium, riboflavin, vitamin B12, folate, zinc, and vitamin A, except for niacin and vitamin B6 across all age groups. Females had lower odds of having higher MPA than males. Adolescents and children under 2 years were less likely to have higher MPA. Participants from large families (>4 members) had lower odds of having higher MPA than those from smaller families.

### Food group intake in the life cycle stage

In the present study, cereals dominated the diet of Bangladeshi people of all life stages. The consumption of pulses, leafy vegetables, nuts and seeds, fruits, and milk was found among a lower percentage of people in all age and vulnerability groups. The inadequate intake of the above-mentioned food groups might lead them to lower intake of different vital micronutrients [29]. Previous studies also highlighted a predominantly cereal-based diet among Bangladeshi adolescents [30], WRA [10,11], pregnant mothers [31], and lactating mothers [22,32]. Their diets are inadequately diverse in most cases, with the absence of leafy vegetables, nuts and seeds, fruits, and milk. However, in the food-based dietary guideline of Bangladesh, it is recommended to consume 1–3 servings of fruits, 3–6 servings of vegetables, 1–2 servings of pulses, and 1–2 servings of milk and milk products daily by an adult person to keep healthy [2]. Similarly, the American Dietary Guidelines 2025–2030 place greater emphasis on high-quality protein sources and healthy fats, with less focus on cereals and refined carbohydrates [33]. Thus, the people of Bangladesh should increase the intake of fruits, vegetables, and animal-source foods rather than heavily relying on the cereals and grains.

Several underlying factors, including the high price of animal-based foods, fruits, etc., low affordability, food insecurity, and lack of awareness, likely contributed to inadequate intake of different food items and led the consumers to cereals-based diets [34,35]. Moreover, 43% of the population in Bangladesh cannot afford a recommended diet due to high costs and food insecurity [34]. Price volatility is another pressing issues in Bangladesh, especially for the lower income groups who are forced to be dependent on cereal-based diets. Sometimes, seasonality also influences the dietary patterns in Banglaesh. Thus, diversification toward pulses, vegetables, and animal‑source foods requires supportive policies on pricing, and availability considering the affordability of the poorer community. Household decision making, food environment, and cultural preferences also influence the dietary choices of the households.

Moreover, in our study, the intake of different food groups increased with age and the highest intake level was observed in the 19–60 age group. After age 60, the intake level of various food groups decreased again. Similarly, increasing consumption trends with age were reported by Loukrakpam et al. [18] among the Northeast Indians. In addition, Wakimoto and Block [36] highlighted a decreasing trend in calorie intake with increasing age groups, and the intake was the lowest among older people (65–74 years). With the advancement of the ageing process, changes in physical abilities and the presence of chronic conditions might influence the dietary pattern and metabolic power of older people [36]. The current study’s findings also highlighted a higher intake of cereals, starchy roots, fish, fats, and oils among lactating mothers compared to WRA and pregnant mothers. However, the intake of non-leafy vegetables and animal-source foods was highest among pregnant women. Previous studies also reported low dietary diversity and inadequate intake of different food groups among WRA [10,11,37].

The cereal-dominant diet may provide energy but can reduce dietary diversity and micronutrient density. The intake of refined grains like polished rice and refined wheat, provide energy but reduce fibre and micronutrient density [38]. The refining process strips away iron, zinc, and B‑vitamins, contributing to hidden hunger despite adequate calorie intake. Low fibre intake from such diets is linked to poor satiety, metabolic risks, and reduced overall diet quality that highlights the importance of diversifying carbohydrate sources. Replacing part of cereal consumption with pulses, vegetables, and animal‑source foods can enhance protein quality, improve micronutrient adequacy, and increase bioavailability of key nutrients such as iron and vitamin B12.

### Nutrient intake and contribution of food group into the macro and micronutrient intake

An increasing trend was found in dietary energy, macro, and micronutrient intake with the participants’ age increase. In addition, our study found the highest median intake of protein, fat, and L-ascorbic acid among pregnant women, while the median intake of energy, carbohydrate, calcium, and magnesium was highest among lactating mothers. On the other hand, a lower intake of energy, macro, and micronutrients was noted among the WRA compared to the pregnant and lactating women. Loukrakpam et al. [18] also found an increasing trend of different nutrient intake with age among the Northeast Indians. Previous studies in Bangladesh also reported a lower intake of energy, macro, and micronutrients among adolescents and WRA [10,13,22]. WRA from neighbouring countries and other low- and middle-income countries also had a lower intake of different micronutrients [18,37,39]. Cereals and cereal-based products mainly contribute to energy and macronutrients. This finding was consistent with previous studies conducted in Bangladesh and other neighbouring countries [10,11,18,37].

Dietary intake and micronutrient adequacy in Bangladesh are shaped by seasonality and local food environments, which often limit dietary diversity and increase reliance on cereal-based staples, particularly during lean periods. While cereals dominate energy intake, they provide limited bioavailable iron and zinc due to the presence of absorption inhibitors such as phytates, compounded by low consumption of enhancers like vitamin C–rich and animal-source foods. Evidence suggests that phytate content in Bangladeshi diet can reduce the bioavalability of iron, and zinc [22]. Consequently, micronutrient inadequacy reflects not only low intake but also poor bioavailability, highlighting the need for context-specific interventions that promote dietary diversification, improved food access, and food fortification while accounting for seasonal variation.

### Probability of nutrient adequacy

The study findings showed median PA was lowest for riboflavin, folate, vitamin B_12_, vitamin C, vitamin A, iron, and calcium in all age and life-stage groups according to the NSB 2017–18 survey and the BIHS 2015 survey. Similar inadequate intake of riboflavin, folate, vitamin B_12_, vitamin A, and calcium was reported in previous studies [10,12,22]. A study among lactating women also concluded low intake of the micronutrients mentioned above [22]. The latest national micronutrient survey of Bangladesh also highlighted deficiencies of different micronutrients among children and women [2]. Inadequate intake of different micronutrient-rich foods, especially leafy vegetables, fruits, dairy products, animal-source foods, etc., might contribute to lower adequacy of multiple micronutrients. Inadequacy of micronutrients might make them vulnerable to developing micronutrient deficiency disorders like anemia. However, the present study observed the highest median PA for niacin, thiamine, and vitamin B6. Several previous studies at national and sub-national levels in Bangladesh also showed comparatively higher adequacy of niacin and vitamin B6 [10,11,22,30]. These vitamins were also found to be adequate among pregnant and lactating women in Bangladesh [22] Moreover, our study findings highlighted that the median pooled MPA of 11 micronutrients among the participants was only 20–30%. This finding was consistent with a study by Arsenault et al. [10] which reported an MPA of 26–43%. The findings thus highlight individuals met the requirements for only about one-third of the assessed micronutrients, reflecting substantial population-level inadequacy. These low MPA levels are consistent with evidence from Bangladesh showing a high prevalence of multiple micronutrient deficiencies, including anemia and inadequate intakes of vitamin A, zinc, calcium, and B vitamins, which are associated with adverse health outcomes such as impaired immunity and poor maternal and child health. From a public health perspective, MPA values well below 50% highlight widespread and concurrent micronutrient gaps, underscoring the need for comprehensive nutrition interventions, including diet diversification, food fortification, and targeted supplementation for vulnerable groups.

### Socio-demographic predictors of the MPA of different micronutrients

The present study findings showed that the females had lower odds of having higher MPA than males. This finding was consistent with previous studies conducted in Bangladesh [40,41]. Intra-household food distribution culture in Bangladesh, especially in rural areas, contributed to the lower intake of food by the women [41]. Moreover, coping strategies against food shortfalls in an insecure family influence the mothers to take the remaining food after feeding all the family members. On the other hand, pre-adolescents and lactating women were more likely to have higher MPA compared to the WRA. Grandner et al. [42] also highlighted an increased consumption of nutrients and calories by women who are pregnant or lactating as opposed to those who are not. However, these women face increased physiological demands for iron, zinc, folate, and vitamin B12, which are often unmet despite sufficient energy intake. Cereal‑dominant diets provide calories but lack diversity, contributing to persistent micronutrient deficiencies and hidden hunger. Limited intake of animal‑source foods further reduces the bioavailability of key nutrients, particularly iron and vitamin B12, exacerbating risks of anemia. In addition, socio‑cultural and intra‑household food allocation practices may disadvantage women, compounding dietary inadequacy. Promoting dietary diversification through pulses, vegetables, and nutrient‑rich foods is therefore essential to improve micronutrient adequacy and address women’s nutrition gaps.

The lower mean probability of adequacy observed among urban participants likely reflects the ongoing dietary transition in Bangladesh. Urban diets are increasingly characterized by higher consumption of processed foods and reduced reliance on traditional staples, which may lower dietary diversity and micronutrient density. Changing affordability patterns and food environments further shape these differences, highlighting the need for policies that promote access to diverse and nutrient‑rich foods in urban settings.

In the BIHS 2015 survey, an increasing trend of MPA was found with higher expenditure quintiles. Previous studies also showed a similar trend with higher income quintiles/classes. Food insecurity leads to low-cost cereal-based dietary intake, causing inadequate intake of different vital nutrients [34,35]. To ensure sufficient intake, the poorer segment of the country has been brought under government incentive policies like open market sales, 30 kg fortified rice under the social safety net program, food for work, vulnerable group feeding, etc. Moreover, the participants from a large family (>4 members) had less chance of having higher MPA than those from a small family. It is supposed that the unaffordability to acquire a sufficient healthy diet in large family, especially in rural areas, might influence their dietary intake and nutrient adequacy.

Although dietary patterns, food environments, and nutrition-related policies in Bangladesh have been changing rapidly, the BIHS 2015 and NSB 2017–18 remain the most recent nationally representative datasets with detailed dietary consumption information. Consequently, the findings of this study should be interpreted as reflecting dietary practices during the respective survey periods rather than current conditions. Nevertheless, these results provide an important baseline against which future changes in food consumption patterns and nutrient adequacy can be assessed. In the absence of more recent nationally representative dietary intake data, the present analysis offers valuable insights into structural gaps in diet quality and nutrient intake that are likely to persist and remain relevant for policy planning. The findings also highlight the urgent need for updated national dietary surveys to inform and guide contemporary nutrition and food policy decisions in Bangladesh.

### Implications of the study findings

The findings underscore a significant micronutrient intake inadequacy across all age, sex, and vulnerability groups in Bangladesh. The low MPA, particularly for calcium, riboflavin, vitamin B12, folate, zinc, and vitamin A, highlights a persistent risk of micronutrient deficiencies in the population. This has important implications for policymakers, health practitioners, and nutrition planners. It signals the need for food and nutrition policies and programs to prioritize increasing the consumption of micronutrient-dense foods, such as pulses, fruits, nuts and seeds, green leafy vegetables, and dairy products. Furthermore, the findings identify several population groups at particularly high risk of micronutrient inadequacy, including children under two years of age, women, especially those of reproductive age, and individuals living in large households. For young children, interventions should prioritize improved complementary feeding practices, access to fortified foods, and caregiver nutrition education. For women, especially adolescents and women of reproductive age, strategies such as large-scale food fortification, strengthened supplementation programs, and behavior change communication to improve diet quality are critical. Households with larger family sizes may benefit from nutrition-sensitive social protection measures, including targeted food assistance and agricultural programs that promote the production and affordability of micronutrient-rich foods. Thus, these findings underscore the need for age-, sex-, and vulnerability-specific policies rather than uniform approaches, to more effectively address the diverse nutritional needs of the Bangladeshi population.

### Strengths and limitations

The present study draws on two large, nationally and rurally representative datasets and applies a comprehensive analytical framework to examine food consumption patterns and nutrient adequacy across lifecycle stages in Bangladesh. A key strength of the study is the use of individual-level dietary intake data from the Nutrition Survey of Bangladesh (NSB) 2017–18, which employed the weighed food record method and included repeated measurements for a subsample of participants, allowing for improved estimation of usual intake. In addition, nutrient adequacy was assessed using life-stage-, sex-, and physiological status–specific Estimated Average Requirement (EAR) values from the ICMR–NIN, enabling a nuanced assessment of both macronutrient and micronutrient adequacy across population subgroups. While Indian EARs provide a practical and regionally appropriate benchmark, alternative standards (e.g., FAO/WHO or other international references) could yield different adequacy estimates.

Despite these strengths, several limitations should be considered when interpreting the findings. First, the two datasets used in this study differ in design and scope. While NSB 2017–18 collected individual-level intake data, the BIHS 2015 relied on 24-hour dietary recall at the household level. Individual intake in BIHS was therefore estimated using the AME method, which assumes proportional intra-household food distribution. Although AME is widely used in large-scale household surveys, it does not capture potential gender- and age-based disparities in food allocation and may lead to measurement error, particularly for women, children, and other vulnerable groups.

Second, the use of 24-hour recall data in BIHS may have introduced recall bias and contributed to both under- and over-estimation of food and nutrient intake. Differences in dietary assessment methods, population coverage (national versus rural), and survey timing between NSB and BIHS limit direct comparability of estimates across datasets. Accordingly, results from each survey should be interpreted within the context of its respective design.

Third, nutrient composition was primarily derived from the FCT for Bangladesh. When nutrient values were unavailable, information was supplemented using the Indian FCT, and for a limited number of items, the USDA database. Although this hierarchical approach follows standard practice in dietary assessment, reliance on international databases may introduce uncertainty, particularly for fortified foods and region-specific varieties. In addition, information on iron–folic acid (IFA) supplementation was uavailable in the secondary datasets used. Consequently, total iron and folate intake may be underestimated, particularly among pregnant and lactating women who may benefit from national supplementation programs. No information was also available for the intake of any iron or other micronutrient-fortified foods. Bioavailability of iron was not considered in our study, therefore, the estimates of adequacy should be interpreted cautiously. In addition, we couldn’t consider the content of phytate and its effect on the bioavailability of iron, zinc, and calcium. Thus, the adequacy level of these nutrients might be overestimated. Future studies incorporating bioavailability, fortification, and supplementation data would provide a more accurate assessment.

Moreover, micronutrient adequacy was assessed using the EAR cut-point method to estimate the PA and MPA. This approach is widely applied in population-level studies. However, it assumes a normal distribution of nutrient requirements and may be less appropriate for nutrients with skewed requirement distributions, such as iron among menstruating women. As a result, iron adequacy estimates should be interpreted with caution.

## Conclusion

Every demographic segment eats much cereal, with rice being the preferred staple cereal across the country, culturally as well as, economically. On the other hand, the intake of fruits, milk and milk products, leafy vegetables, nuts, and seeds was low among different sub-groups. Except for niacin and vitamin B_6_, the calcium, riboflavin, thiamine, vitamin B_12_, and vitamin A intake were critically inadequate. Compared to men, women had lower levels of adequacy. The level of nutrient adequacy rises with age. The MPA of the micronutrients was low among all ages and physiological stage groups. The females, participants from large families, children under two years, pre-adolescents, and WRA were at higher risk of nutrient inadequacy. Increased consumption of pulses, fruits, nuts and seeds, vegetables (particularly leafy vegetables), and dairy products will help to address the micronutrient gap. Policies and strategies should promote the production, affordability, accessibility, and consumption of nutrient-dense foods, particularly pulses, fruits, nuts and seeds, leafy vegetables, and dairy products, to improve micronutrient adequacy.

## Supporting information

S1 File Supplementary Tables and Figures. (DOCX)
