# Supplementary material for: Food consumption patterns and nutrient adequacy across lifecycle stages in Bangladesh
Source: PLoS One. 2026 Sep 28;21(9):e0359205. doi: 10.1371/journal.pone.0359205 (PMC13618963; doi:10.1371/journal.pone.0359205)
Supplement: Supplementary file 2 [file pone.0359205.s004.pdf]

Reviewer 2 comments on Revised version of the manuscript

I observed some key improvements:

1. **Methodological Rigor:** The authors clearly justified using both NSB and BIHS datasets, detailed how usual intake was calculated using variance decomposition, and explicitly stated the limitations of using the Adult Male Equivalent (AME) method and Indian EAR values.
2. **Nutritional Depth:** The discussion was substantially expanded to cover carbohydrate quality (polished rice vs. fiber/whole grains), antinutritional factors (phytates reducing iron and zinc bioavailability), hidden hunger in women of reproductive age, and the impact of urban dietary transitions.
3. **Statistical & Visual Transparency:** Model assumptions for the multivariate logistic regression (VIF, variable selection, MPA > 0.5 threshold) were fully specified, and Table 3 and p-value formatting issues were corrected.

Recommendation: Accept. Every critique was directly integrated into both the manuscript text and the limitations section, resulting in a much stronger, scientifically sound paper ready for publication.
