# Supplementary material for: Food consumption patterns and nutrient adequacy across lifecycle stages in Bangladesh
Source: PLoS One. 2026 Sep 28;21(9):e0359205. doi: 10.1371/journal.pone.0359205 (PMC13618963; doi:10.1371/journal.pone.0359205)
Supplement: Supplementary file 3 [file pone.0359205.s002.pdf]

This manuscript addresses an important issue related to dietary patterns and micronutrient adequacy across different lifecycle stages in Bangladesh. Using NSB 2017–18 and BIHS 2015 datasets, the authors apply PA and MPA approaches to assess nutrient gaps.

The study highlights a highly cereal-based dietary pattern with limited intake of diverse food groups such as fruits, vegetables, pulses, nuts/seeds, and dairy products. The findings related to inadequate calcium, riboflavin, vitamin B12, folate, zinc, and vitamin A intake are relevant not only for Bangladesh but also for broader South Asian nutrition contexts where similar dietary transitions and food access challenges exist.

The manuscript has strengths including large datasets, inclusion of different lifecycle groups, and use of nutrient adequacy methods. However, several points require clarification and deeper interpretation.

Recommendation: Major Revision

1. The authors should clarify the purpose of using NSB and BIHS datasets and explain why these sources were selected. Since the surveys differ in dietary assessment methods, population coverage, and analytical approach, results should not appear directly comparable. A clear comparison table describing survey design, dietary method, sample characteristics, and limitations would improve the manuscript.
2. More methodological details are needed regarding usual intake estimation. The authors should explain how intra- and inter-individual variation was adjusted, whether repeated dietary measurements were available, and how this may influence nutrient adequacy estimates.
3. The use of Adult Male Equivalent (AME) requires more discussion. In South Asian households, food distribution is often influenced by gender, age, household role, and social factors. The authors should acknowledge how AME assumptions may affect estimates among women and children, particularly vulnerable groups.
4. The selection of ICMR-NIN Indian EAR values requires stronger justification. Although India and Bangladesh have similar dietary patterns and regional characteristics, differences in population, nutritional status, and requirements should be considered. The authors should discuss whether using alternative standards could change conclusions.
5. The reported iron adequacy among pregnant and lactating women requires careful interpretation. Extremely high adequacy estimates appear inconsistent with the known burden of anemia in Bangladesh and South Asia. The authors should discuss iron supplementation, dietary bioavailability, inclusion of fortified foods, and limitations of the EAR cut-point approach for women with increased physiological requirements.
6. The manuscript should provide greater attention to the quality of carbohydrate sources rather than only quantity. A diet dominated by cereals may provide energy but can reduce dietary diversity and micronutrient density. The discussion should consider the role of refined grains, dietary fibre, and replacement of cereals with pulses, vegetables, and animal-source foods.
7. Since cereals contribute substantially to energy and micronutrient intake, the authors should discuss antinutritional factors such as phytate, which can reduce absorption of iron

and zinc. Nutrient intake estimates should be interpreted alongside considerations of nutrient bioavailability.

8. The discussion should further consider women's nutrition, particularly women of reproductive age. Adequate energy intake does not necessarily indicate adequate nutrition, and micronutrient deficiencies may persist despite sufficient calorie consumption.
9. Differences in dietary patterns across urban and rural settings require deeper interpretation. The finding of lower MPA among urban participants may reflect dietary transition, increased consumption of processed foods, reduced traditional diets, or changing food affordability patterns.
10. The regression analysis requires additional information on survey weighting, clustering, variable selection, model assumptions, and justification for using MPA >0.5 as the outcome definition.
11. The manuscript would benefit from a stronger discussion of economic and food system factors. Dietary choices in Bangladesh, as in many South Asian countries, are influenced not only by awareness but also by income, food prices, availability, cultural preferences, and household decision-making.
12. The abstract should be shortened and focused on the main findings. Terminology should be standardized throughout. Vitamin B12 units in Table 3 should be corrected to µg, p-values should be reported as $p < 0.001$ rather than 0.000, and language editing is recommended.

Overall, this manuscript provides valuable evidence on dietary diversity and micronutrient inadequacy in Bangladesh. With clearer methodology, improved discussion of regional dietary patterns, and more cautious interpretation of nutrient adequacy findings, the study could make an important contribution to nutrition research in South Asia.
