# Supplementary material for: Food consumption patterns and nutrient adequacy across lifecycle stages in Bangladesh
Source: PLoS One. 2026 Sep 28;21(9):e0359205. doi: 10.1371/journal.pone.0359205 (PMC13618963; doi:10.1371/journal.pone.0359205)
Supplement: Supplementary file 3 [file pone.0359205.s001.docx]

Supporting Information

Supplementary Table 1: Key features of the Nutrition Survey of Bangladesh (NSB 2017–18) and Bangladesh Integrated Household Survey (BIHS 2015)

| **Feature** | **NSB 2017–18** | **BIHS 2015** |
| --- | --- | --- |
| Implementing agency | Institute of Nutrition and Food Science, University of Dhaka | International Food Policy Research Institute (IFPRI) |
| Survey design | Nationally representative, stratified sampling with PPS | Nationally representative rural panel survey, two-stage stratified random sampling |
| Population coverage | Rural and urban, all divisions; oversampling of urban households | Rural households only |
| Sample size | 3,541 individuals | 5,503 households (~13,000 individuals) |
| Dietary assessment method | Weighed food record (individual-level) | Two non-consecutive 24-hour recalls (individual-level) + household food acquisition |
| Strengths | Detailed nutrient intake, nationally representative | Usual intake estimation, socioeconomic linkages |
| Limitations | Limited to one survey round, food weighed record might cause high respondent burden, behavioral changes, practical difficulties in social settings, and challenges in capturing meals consumed away from home | Coverage of rural areas only, potential recall bias |

|  | Cereals | Pulses | Non-leafy vegetables | Leafy vegetables | Starchy roots | Fruits | Fish | Meat and Egg | Milk and milk product | Fats and oils | Spices | Others |
| --- | --- | --- | --- | --- | --- | --- | --- | --- | --- | --- | --- | --- |
| Energy | 69.823% | 2.24198% | 2.43223% | 0.299271% | 2.35265% | 0.471743% | 3.55576% | 2.26497% | 1.13834% | 13.6971% | 0.719167% | 1% |
| Protein | 53.37% | 6.39977% | 4.21579% | 1.02261% | 1.71686% | 0.250695% | 18.3837% | 11.2639% | 1.96719% | 0.0001382% | 0.0018117% | 1.4% |
| Carbohydrate | 89.7459% | 1.90551% | 2.4488% | 0.142761% | 2.88406% | 0.540881% | 4.42e-05% | 0.0065656% | 0.568096% | 2.14e-06% | 1.06626% | 0.7% |
| Fat | 6.63198% | 0.53175% | 0.509955% | 0.241325% | 0.337769% | 0.214978% | 8.22815% | 4.44249% | 3.04955% | 74.223% | 0.0004437% | 1.6% |

Supplementary Figure 1: Food group’s supply to the daily intake of selected macronutrients in 2017-18

Percentage

|  | Cereals | Pulses | Vegetables | Leafy Vegetables | Starchy roots | Fruits | Fish | Meat and Egg | Milk and Milk product | Edible oils | Spices | Others |
| --- | --- | --- | --- | --- | --- | --- | --- | --- | --- | --- | --- | --- |
| Calcium | 22.3772 | 2.39451 | 15.4002 | 6.89724 | 4.88073 | 0.798764 | 21.1906 | 4.21558 | 11.8731 | 0.0028196 | 0.3 | 9.66935 |
| Iron | 44.34 | 7.71598 | 12.2169 | 4.53794 | 4.69681 | 0.59158 | 7.14432 | 5.45863 | 0.383744 | 0.184167 | 0.8 | 11.93 |
| Vitamin A (RAE) | 0.0611612 | 1.37631 | 16.1264 | 12.422 | 3.52729 | 1.72169 | 11.9815 | 23.5237 | 10.5989 | 0.200184 | 1.5 | 16.9608 |
| Folate | 32.3863 | 4.47209 | 17.1838 | 6.74738 | 8.63797 | 1.48038 | 19.5403 | 5.77694 | 1.41655 | 0.0002887 | 0.4 | 1.95797 |

Supplementary Figure 2: Food group’s supply to the daily intake of selected micronutrients in 2017-18
