# Supplementary material for: Food consumption patterns and nutrient adequacy across lifecycle stages in Bangladesh
Source: PLoS One. 2026 Sep 28;21(9):e0359205. doi: 10.1371/journal.pone.0359205 (PMC13618963; doi:10.1371/journal.pone.0359205)
Supplement: Peer review history

# Peer review: Decision Letter 0

**DOI:** 10.1371/journal.pone.0359205.r001

**Authors:** Zehra Batu

22 Jun 2026

Dear Dr. Amin,

Thank you for submitting your manuscript to PLOS ONE. After careful consideration, we feel that it has merit but does not fully meet PLOS ONE’s publication criteria as it currently stands. Therefore, we invite you to submit a revised version of the manuscript that addresses the points raised during the review process.

===========================

Please fully review Reviewer 2's feedback and respond to all suggestions.

===========================

Please submit your revised manuscript by Aug 21 2026 11:59PM. If you will need more time than this to complete your revisions, please reply to this message or contact the journal office at plosone@plos.org. When you're ready to submit your revision, log on to https://www.editorialmanager.com/pone/ and select the 'Submissions Needing Revision' folder to locate your manuscript file.

- A letter that responds to each point raised by the academic editor and reviewer(s). You should upload this letter as a separate file labeled 'Response to Reviewers'.
- A marked-up copy of your manuscript that highlights changes made to the original version. You should upload this as a separate file labeled 'Revised Manuscript with Track Changes'.
- An unmarked version of your revised paper without tracked changes. You should upload this as a separate file labeled 'Manuscript'.

If you would like to make changes to your financial disclosure, please include your updated statement in your cover letter. Guidelines for resubmitting your figure files are available below the reviewer comments at the end of this letter.

If applicable, we recommend that you deposit your laboratory protocols in protocols.io to enhance the reproducibility of your results. Protocols.io assigns your protocol its own identifier (DOI) so that it can be cited independently in the future. For instructions see: https://journals.plos.org/plosone/s/submission-guidelines#loc-laboratory-protocols. Additionally, PLOS ONE offers an option for publishing peer-reviewed Lab Protocol articles, which describe protocols hosted on protocols.io. Read more information on sharing protocols at https://plos.org/protocols?utm_medium=editorial-email&utm_source=authorletters&utm_campaign=protocols.

As the corresponding author, your ORCID iD is verified in the submission system and will appear in the published article. PLOS supports the use of ORCID, and we encourage all coauthors to register for an ORCID iD and use it as well. Please encourage your coauthors to verify their ORCID iD within the submission system before final acceptance, as unverified ORCID iDs will not appear in the published article. *Only*  the individual author can complete the verification step; PLOS staff *cannot*  verify ORCID iDs on behalf of authors.

We look forward to receiving your revised manuscript.

Kind regards,

Zehra Batu

Academic Editor

PLOS One

Journal Requirements:

When submitting your revision, we need you to address these additional requirements.

1. Please ensure that your manuscript meets PLOS ONE's style requirements, including those for file naming. The PLOS ONE style templates can be found at

https://journals.plos.org/plosone/s/file?id=wjVg/PLOSOne_formatting_sample_main_body.pdf and https://journals.plos.org/plosone/s/file?id=ba62/PLOSOne_formatting_sample_title_authors_affiliations.pdf

2. Your ethics statement should only appear in the Methods section of your manuscript. If your ethics statement is written in any section besides the Methods, please delete it from any other section.

3. Please include captions for your Supporting Information files at the end of your manuscript, and update any in-text citations to match accordingly. Please see our Supporting Information guidelines for more information: http://journals.plos.org/plosone/s/supporting-information.

4. If the reviewer comments include a recommendation to cite specific previously published works, please review and evaluate these publications to determine whether they are relevant and should be cited. There is no requirement to cite these works unless the editor has indicated otherwise.

[Note: HTML markup is below. Please do not edit.]

Reviewers' comments:

Reviewer's Responses to Questions

**Comments to the Author**

1. Is the manuscript technically sound, and do the data support the conclusions?

Reviewer #1: Yes

Reviewer #2: Yes

**********

2. Has the statistical analysis been performed appropriately and rigorously?

Reviewer #1: Yes

Reviewer #2: Yes

**********

3. Have the authors made all data underlying the findings in their manuscript fully available?

The PLOS Data policy

Reviewer #1: Yes

Reviewer #2: Yes

**********

4. Is the manuscript presented in an intelligible fashion and written in standard English?

Reviewer #1: Yes

Reviewer #2: Yes

**********

Reviewer #1: An interesting work which highlights the need for conducting nutrition surveys in order to rethink nutrition programmes in low and middle-resources countries according to local and cultural particularities. The manuscript is written in a clear and understandable manner. The statistical analyses are detailed and explained.

Reviewer #2: This manuscript addresses an important issue related to dietary patterns and micronutrient

adequacy across different lifecycle stages in Bangladesh. Using NSB 2017–18 and BIHS 2015

datasets, the authors apply PA and MPA approaches to assess nutrient gaps.

The study highlights a highly cereal-based dietary pattern with limited intake of diverse food

groups such as fruits, vegetables, pulses, nuts/seeds, and dairy products. The findings related to

inadequate calcium, riboflavin, vitamin B12, folate, zinc, and vitamin A intake are relevant not

only for Bangladesh but also for broader South Asian nutrition contexts where similar dietary

transitions and food access challenges exist.

The manuscript has strengths including large datasets, inclusion of different lifecycle groups, and

use of nutrient adequacy methods. However, several points require clarification and deeper

interpretation.

Recommendation: Major Revision

1. The authors should clarify the purpose of using NSB and BIHS datasets and explain why

these sources were selected. Since the surveys differ in dietary assessment methods,

population coverage, and analytical approach, results should not appear directly

comparable. A clear comparison table describing survey design, dietary method, sample

characteristics, and limitations would improve the manuscript.

2. More methodological details are needed regarding usual intake estimation. The authors

should explain how intra- and inter-individual variation was adjusted, whether repeated

dietary measurements were available, and how this may influence nutrient adequacy

estimates.

3. The use of Adult Male Equivalent (AME) requires more discussion. In South Asian

households, food distribution is often influenced by gender, age, household role, and social

factors. The authors should acknowledge how AME assumptions may affect estimates

among women and children, particularly vulnerable groups.

4. The selection of ICMR-NIN Indian EAR values requires stronger justification. Although

India and Bangladesh have similar dietary patterns and regional characteristics, differences

in population, nutritional status, and requirements should be considered. The authors

should discuss whether using alternative standards could change conclusions.

5. The reported iron adequacy among pregnant and lactating women requires careful

interpretation. Extremely high adequacy estimates appear inconsistent with the known

burden of anemia in Bangladesh and South Asia. The authors should discuss iron

supplementation, dietary bioavailability, inclusion of fortified foods, and limitations of the

EAR cut-point approach for women with increased physiological requirements.

6. The manuscript should provide greater attention to the quality of carbohydrate sources

rather than only quantity. A diet dominated by cereals may provide energy but can reduce

dietary diversity and micronutrient density. The discussion should consider the role of

refined grains, dietary fibre, and replacement of cereals with pulses, vegetables, and

animal-source foods.

7. Since cereals contribute substantially to energy and micronutrient intake, the authors

should discuss antinutritional factors such as phytate, which can reduce absorption of iron

and zinc. Nutrient intake estimates should be interpreted alongside considerations of

nutrient bioavailability.

8. The discussion should further consider women’s nutrition, particularly women of

reproductive age. Adequate energy intake does not necessarily indicate adequate nutrition,

and micronutrient deficiencies may persist despite sufficient calorie consumption.

9. Differences in dietary patterns across urban and rural settings require deeper interpretation.

The finding of lower MPA among urban participants may reflect dietary transition,

increased consumption of processed foods, reduced traditional diets, or changing food

affordability patterns.

10. The regression analysis requires additional information on survey weighting, clustering,

variable selection, model assumptions, and justification for using MPA >0.5 as the

outcome definition.

11. The manuscript would benefit from a stronger discussion of economic and food system

factors. Dietary choices in Bangladesh, as in many South Asian countries, are influenced

not only by awareness but also by income, food prices, availability, cultural preferences,

and household decision-making.

12. The abstract should be shortened and focused on the main findings. Terminology should

be standardized throughout. Vitamin B12 units in Table 3 should be corrected to µg, p-

values should be reported as p < 0.001 rather than 0.000, and language editing is

recommended.

Overall, this manuscript provides valuable evidence on dietary diversity and micronutrient

inadequacy in Bangladesh. With clearer methodology, improved discussion of regional dietary

patterns, and more cautious interpretation of nutrient adequacy findings, the study could make an

important contribution to nutrition research in South Asia.

**********

what does this mean?). If published, this will include your full peer review and any attached files.

If you choose “no”, your identity will remain anonymous but your review may still be made public.

**Do you want your identity to be public for this peer review?**  For information about this choice, including consent withdrawal, please see our Privacy Policy

Reviewer #1: No

Reviewer #2: No

**********

[NOTE: If reviewer comments were submitted as an attachment file, they will be attached to this email and accessible via the submission site. Please log into your account, locate the manuscript record, and check for the action link "View Attachments". If this link does not appear, there are no attachment files.]

To ensure your figures meet our technical requirements, please review our figure guidelines: https://journals.plos.org/plosone/s/figures

You may also use PLOS’s free figure tool, NAAS, to help you prepare publication quality figures: https://journals.plos.org/plosone/s/figures#loc-tools-for-figure-preparation.

NAAS will assess whether your figures meet our technical requirements by comparing each figure against our figure specifications.

Attachment Submitted filename: Plos one -Nutrition_Bdesh..pdf

# Author comment: Author response to Decision Letter 1

**DOI:** 10.1371/journal.pone.0359205.r002

19 Aug 2026

Authors’ response to reviewers

Reviewer #1

Comments: An interesting work which highlights the need for conducting nutrition surveys in order to rethink nutrition programmes in low and middle-resources countries according to local and cultural particularities. The manuscript is written in a clear and understandable manner. The statistical analyses are detailed and explained.

Response: Thank you.

Reviewer #2

Comment 1. The authors should clarify the purpose of using NSB and BIHS datasets and explain why these sources were selected. Since the surveys differ in dietary assessment methods, population coverage, and analytical approach, results should not appear directly comparable. A clear comparison table describing survey design, dietary method, sample characteristics, and limitations would improve the manuscript.

Response: We appreciate the reviewer’s observation regarding the use of NSB 2017–18 and BIHS 2015 datasets. Our intention in employing both surveys was not to directly compare results across them, but rather to leverage their complementary strengths in assessing food consumption and nutrient adequacy across lifecycle stages in Bangladesh. The NSB provides nationally representative, individual-level weighed food records with detailed nutrient intake data, while the BIHS offers household-level food acquisition and consumption data linked to socioeconomic characteristics, including repeated 24-hour recalls that allow estimation of usual intake. By analyzing these datasets separately, we aimed to generate a more comprehensive picture of dietary patterns and nutrient adequacy.

We agree that the differences in dietary assessment methods, population coverage, and analytical approaches should be made explicit to avoid any impression of direct comparability. To address this, we have added a comparison table (supplementary Table 1) summarizing the key features of each survey, including survey design, dietary assessment method, sample characteristics, and limitations.

Please see the lines 115-128 and supplementary Table 1.

Comment 2. More methodological details are needed regarding usual intake estimation. The authors should explain how intra- and inter-individual variation was adjusted, whether repeated dietary measurements were available, and how this may influence nutrient adequacy estimates.

Response: We thank the reviewer for this important comment. We have revised the Methods section to clarify that usual intake was estimated using repeated dietary measures and adjusted for intra- and inter-individual variation with log-transformed data and variance decomposition. We also note that both NSB 2017–18 and BIHS 2015 included repeated dietary measurements, which improved estimation of nutrient adequacy, while unadjusted within-person variation may overestimate inadequacy.

See the clarification in lines 206-212 in the methodology section

“The usual intake was estimated by adjusting for intra- and inter-individual variation using log-transformed intake data and ANOVA-based variance decomposition, followed by back-transformation to derive habitual intake distributions; repeated dietary measurements were available in BIHS 2015, which allowed for correction of within-person variation and improved estimation of nutrient adequacy, as unadjusted intake data can overestimate the prevalence of inadequacy due to day-to-day variability.”

Comment 3. The use of Adult Male Equivalent (AME) requires more discussion. In South Asian households, food distribution is often influenced by gender, age, household role, and social factors. The authors should acknowledge how AME assumptions may affect estimates among women and children, particularly vulnerable groups.

Response: We appreciate the reviewer’s concern regarding the Adult Male Equivalent (AME) method. AME was applied only to BIHS data to individualize household food and nutrient consumption, following established practice in Bangladesh (Waid et al., 2017). While this approach assumes equal‑share allocation, we acknowledge that it cannot capture gender‑, age‑, or role‑based disparities in intra‑household food distribution. Estimates for women, children, and vulnerable groups may therefore be affected. We have expanded the Methods and Limitations sections to clarify these assumptions and emphasize cautious interpretation, noting the need for future research using individual‑level intake data to validate AME‑based estimates.

See the clarification in lines 177-185 in the methodology section

“The adult male equivalent (AME) was used to individualize household food and nutrient consumption from the BIHS dataset based on age, and sex of household members. This approach allows for a more accurate estimation of per capita food intake, assuming equal distribution among all household members. The datasets were cleaned, processed, shaped, and combined to create the final analytical dataset. This study employed the same AME as previous research conducted in Bangladesh (Waid et al., 2017). While this approach improves upon equal‑share allocation, we acknowledge that it cannot capture gender‑, age‑, or role‑based disparities in intra‑household food distribution. Estimates for women, children, and vulnerable groups may therefore be affected.”

Also, see the lines 565-569 in the limitation sections.

“Individual intake in BIHS was therefore estimated using the AME method, which assumes proportional intra-household food distribution. Although AME is widely used in large-scale household surveys, it does not capture potential gender- and age-based disparities in food allocation and may lead to measurement error, particularly for women, children, and other vulnerable groups.”

Comment 4. The selection of ICMR-NIN Indian EAR values requires stronger justification. Although India and Bangladesh have similar dietary patterns and regional characteristics, differences in population, nutritional status, and requirements should be considered. The authors should discuss whether using alternative standards could change conclusions.

Response: We acknowledge the reviewer’s concern regarding the selection of ICMR‑NIN Indian Estimated Average Requirement (EAR) values. These were chosen because Bangladesh does not yet have its own nationally endorsed nutrient requirement standards, and the Indian EARs are regionally relevant, reflecting similar dietary patterns, food systems, and environmental exposures. Moreover, the food-based dietary guidelines of Bangladesh also report these ICMR‑NIN Indian EAR values. We agree, however, that differences in population characteristics, nutritional status, and physiological requirements between India and Bangladesh may influence estimates. To address this, we have clarified in the Methods and Limitations sections that while Indian EARs provide a practical and regionally appropriate benchmark, alternative standards (e.g., FAO/WHO or other international references) could yield different adequacy estimates. We emphasize that our conclusions should be interpreted in light of this limitation and highlight the need for Bangladesh‑specific nutrient requirement standards to strengthen future analyses.

See the lines 200-206 in the methodology section

“The National Institute of Nutrition, India's estimated average requirements (EARs) were used to assess the probability of adequacy (PA) of the 11 micronutrients [26]. These ICMR‑NIN Indian EAR values were chosen because Bangladesh does not yet have its own nationally endorsed nutrient requirement standards, and the Indian EARs are regionally relevant, reflecting similar dietary patterns, food systems, and environmental exposures. Moreover, the food-based dietary guidelines of Bangladesh also report these ICMR‑NIN Indian EAR values. We determined the average intake to assess each person's adequacy of dietary intake.”

See the lines 555-561 in the limitation section

“In addition, nutrient adequacy was assessed using life-stage-, sex-, and physiological status–specific Estimated Average Requirement (EAR) values from the ICMR–NIN, enabling a nuanced assessment of both macronutrient and micronutrient adequacy across population subgroups. While Indian EARs provide a practical and regionally appropriate benchmark, alternative standards (e.g., FAO/WHO or other international references) could yield different adequacy estimates.”

Comment 5. The reported iron adequacy among pregnant and lactating women requires careful interpretation. Extremely high adequacy estimates appear inconsistent with the known burden of anemia in Bangladesh and South Asia. The authors should discuss iron supplementation, dietary bioavailability, inclusion of fortified foods, and limitations of the EAR cut-point approach for women with increased physiological requirements.

Response: We appreciate the reviewer’s concern regarding the high iron adequacy estimates among pregnant and lactating women. We agree that these findings should be interpreted with caution given the well‑documented burden of anemia in Bangladesh and South Asia. As already mentioned in our Limitations, information on iron–folic acid (IFA) supplementation was unavailable in the secondary datasets, which may lead to underestimation of total intake. Moreover, the EAR cut‑point method assumes a normal distribution of requirements and is less appropriate for nutrients like iron, where physiological needs vary widely. We have expanded the discussion to highlight that factors such as supplementation programs, dietary bioavailability, and fortified foods may influence adequacy estimates. Future studies incorporating biomarkers and supplementation data would provide a more accurate assessment.

See lines 450-457

“While cereals dominate energy intake, they provide limited bioavailable iron and zinc due to the presence of absorption inhibitors such as phytates, compounded by low consumption of enhancers like vitamin C–rich and animal-source foods. Evidence suggests that phytate content in Bangladeshi diet can reduce the bio-availability of iron, zinc and calcium. Consequently, micronutrient inadequacy reflects not only low intake but also poor bioavailability, highlighting the need for context-specific interventions that promote dietary diversification, improved food access, and food fortification while accounting for seasonal variation.”

In the limitation section, we have mentioned “In addition, nutrient adequacy was assessed using life-stage-, sex-, and physiological status–specific Estimated Average Requirement (EAR) values from the ICMR–NIN, enabling a nuanced assessment of both macronutrient and micronutrient adequacy across population subgroups. While Indian EARs provide a practical and regionally appropriate benchmark, alternative standards (e.g., FAO/WHO or other international references) could yield different adequacy estimates.”

Also see the lines 579-593

“In addition, information on iron–folic acid (IFA) supplementation was unavailable in the secondary datasets used. Consequently, total iron and folate intake may be underestimated, particularly among pregnant and lactating women who may benefit from national supplementation programs. No information was also available for the intake of any iron or other micronutrient-fortified foods. Bioavailability of iron was not considered in our study, therefore, the estimates of adequacy should be interpreted cautiously. In addition, we couldn’t consider the content of phytate and its effect on the bioavailability of iron, zinc, and calcium. Thus, the adequacy level of these nutrients might be overestimated. Future studies incorporating bioavailability, fortification, and supplementation data would provide a more accurate assessment.

Moreover, micronutrient adequacy was assessed using the EAR cut-point method to estimate the PA and MPA. This approach is widely applied in population-level studies. However, it assumes a normal distribution of nutrient requirements and may be less appropriate for nutrients with skewed requirement distributions, such as iron among menstruating women. As a result, iron adequacy estimates should be interpreted with caution.”

Comment 6. The manuscript should provide greater attention to the quality of carbohydrate sources rather than only quantity. A diet dominated by cereals may provide energy but can reduce dietary diversity and micronutrient density. The discussion should consider the role of refined grains, dietary fibre, and replacement of cereals with pulses, vegetables, and animal-source foods.

Response: We thank the reviewer for emphasizing the importance of carbohydrate quality. In our revised Discussion, we now highlight that while cereals remain the dominant energy source in Bangladeshi diets, reliance on refined grains can reduce dietary diversity and micronutrient density. We have added text mentioning the role of dietary fibre and the potential benefits of replacing part of cereal intake with pulses, vegetables, and animal‑source foods to improve nutrient adequacy.

Please see the lines 425-433 as follows

“The cereal-dominant diet may provide energy but can reduce dietary diversity and micronutrient density. The intake of refined grains like polished rice and refined wheat, provide energy but reduce fibre and micronutrient density. The refining process strips away iron, zinc, and B‑vitamins, contributing to hidden hunger despite adequate calorie intake. Low fibre intake from such diets is linked to poor satiety, metabolic risks, and reduced overall diet quality that highlights the importance of diversifying carbohydrate sources. Replacing part of cereal consumption with pulses, vegetables, and animal‑source foods can enhance protein quality, improve micronutrient adequacy, and increase bioavailability of key nutrients such as iron and vitamin B12.”

Comment 7. Since cereals contribute substantially to energy and micronutrient intake, the authors should discuss antinutritional factors such as phytate, which can reduce absorption of iron and zinc. Nutrient intake estimates should be interpreted alongside considerations of nutrient bioavailability.

Response: We thank the reviewer for raising the issue of antinutritional factors. We agree that while cereals contribute substantially to energy and micronutrient intake, high phytate content can inhibit absorption of key minerals such as iron and zinc. In the revised Discussion, we have noted that nutrient intake estimates should be interpreted alongside considerations of bioavailability, particularly in cereal‑based diets. We emphasize that the apparent adequacy of iron and zinc may be overestimated if phytate levels are not accounted for, and highlight the importance of dietary diversification, including pulses, vegetables, and animal‑source food, to improve both intake and absorption.

Please see the lines 448-457 as follows

“Dietary intake and micronutrient adequacy in Bangladesh are shaped by seasonality and local food environments, which often limit dietary diversity and increase reliance on cereal-based staples, particularly during lean periods. While cereals dominate energy intake, they provide limited bioavailable iron and zinc due to the presence of absorption inhibitors such as phytates, compounded by low consumption of enhancers like vitamin C–rich and animal-source foods. Evidence suggests that phytate content in Bangladeshi diet can reduce the bio-availability of iron, zinc and calcium. Consequently, micronutrient inadequacy reflects not only low intake but also poor bioavailability, highlighting the need for context-specific interventions that promote dietary diversification, improved food access, and food fortification while accounting for seasonal variation.”

Comment 8. The discussion should further consider women’s nutrition, particularly women of reproductive age. Adequate energy intake does not necessarily indicate adequate nutrition, and micronutrient deficiencies may persist despite sufficient calorie consumption.

We appreciate the reviewer’s suggestion to expand the discussion on women’s nutrition. In the revised manuscript, we emphasize that adequate energy intake does not necessarily equate to adequate nutrition for women of reproductive age. Micronutrient deficiencies (particularly iron, zinc, folate, and vitamin B12) may persist despite sufficient calorie consumption. We have added discussion highlighting that women’s increased physiological requirements, combined with dietary patterns dominated by cereals, place them at higher risk of hidden hunger. Also, we have mentioned the importance of dietary diversity and nutrient-rich foods.

Please see the lines 485-501

“The present study findings showed that the females had lower odds of having higher MPA than males. This finding was consistent with previous studies conducted in Bangladesh [37,38]. Intra-household food distribution culture in Bangladesh, especially in rural areas, contributed to the lower intake of food by the women [38]. Moreover, coping strategies against food shortfalls in an insecure family influence the mothers to take the remaining food after feeding all the family members. On the other hand, pre-adolescents and lactating women were more likely to have higher MPA compared to the WRA. Grandner et al. [39] also highlighted an increased consumption of nutrients and calories by women who are pregnant or lactating as opposed to those who are not. However, these women face increased physiological demands for iron, zinc, folate, and vitamin B12, which are often unmet despite sufficient energy intake. Cereal‑dominant diets provide calories but lack diversity, contributing to persistent micronutrient deficiencies and hidden hunger. Limited intake of animal‑source foods further reduces the bioavailability of key nutrients, particularly iron and vitamin B12, exacerbating risks of anemia. In addition, socio‑cultural and intra‑household food allocation practices may disadvantage women, compounding dietary inadequacy. Promoting dietary diversification through pulses, vegetables, and nutrient‑rich foods is therefore essential to improve micronutrient adequacy and address women’s nutrition gaps.”

Comment 9. Differences in dietary patterns across urban and rural settings require deeper interpretation.

The finding of lower MPA among urban participants may reflect dietary transition, increased consumption of processed foods, reduced traditional diets, or changing food affordability patterns.

Response: We thank the reviewer for highlighting the need for more interpretation of urban–rural dietary differences. In the revised Discussion, we emphasize that the lower mean probability of adequacy (MPA) observed among urban participants likely reflects ongoing dietary transition. Urban diets are increasingly characterized by higher consumption of processed foods, reduced reliance on traditional diets, and changing affordability patterns. These shifts may reduce dietary diversity and micronutrient density despite adequate energy intake.

Please see the lines 502-507 in the revised manuscript.

We have added following text in the revised manuscript.

“The lower mean probability of adequacy observed among urban participants likely reflects the ongoing dietary transition in Bangladesh. Urban diets are increasingly characterized by higher consumption of processed foods and reduced reliance on traditional staples, which may lower dietary diversity and micronutrient density. Changing affordability patterns and food environments further shape these differences, highlighting the need for policies that promote access to diverse and nutrient‑rich foods in urban settings.”

Response 10. The regression analysis requires additional information on survey weighting, clustering, variable selection, model assumptions, and justification for using MPA >0.5 as the outcome definition.

Response: We thank the reviewer for pointing out the need for greater detail on regression methods. In the revised Methods section, we have clarified that analyses incorporated variable selection, regression analysis assumptions, etc. Regarding the outcome definition, we selected MPA > 0.5 as a pragmatic threshold to indicate that at least half of the micronutrients met adequacy, consistent with prior literature. We have emphasized that this cut‑point is an operational choice and that alternative thresholds could be explored in future work.

Please find the lines 234-240 as follows

“The study employed multivariate logistic regression analysis to determine the parameters linked to nutrient adequacy (MPA > 0.5). This cut‑point is an operational choice supported by prior literature, and alternative thresholds could be explored in future research. The variable selection was guided by the threshold p < 0.2 in the univariate analysis. Multicollinearity among the independent variables was assessed, and a variance inflation factor (VIF) of less than 10 was considered to indicate the absence of multicollinearity. The level of significance was considered at p < 0.05.”

Comment 11. The manuscript would benefit from a stronger discussion of economic and food system factors. Dietary choices in Bangladesh, as in many South Asian countries, are influenced not only by awareness but also by income, food prices, availability, cultural preferences, and household decision-making.

Response: We thank the reviewer for highlighting the importance of economic and food system determinants of diet. In the revised Discussion, we now emphasize that dietary choices in Bangladesh are shaped not only by awareness but also by income, food prices, availability, cultural preferences, and household decision‑making. Our expenditure quintile analysis shows clear socioeconomic gradients in nutrient adequacy, with higher quintiles consistently associated with improved diet quality and adequacy. This highlights that affordability and access remain critical barriers for lower‑income households. We have added text mentioning that cereal‑dominant diets among poorer groups reflect both economic constraints and diversification toward pulses, vegetables, and animal‑source foods require supportive policies on pricing, availability, and social norms.

Please find the lines 402-412 as follows

“Several underlying factors, including the high price of animal-based foods, fruits, etc., low affordability, food insecurity, and lack of awareness, likely contributed to inadequate intake of different food items and led the consumers to cereals-based diets [31,32]. Moreover, 43% of the population in Bangladesh cannot afford a recommended diet due to high costs and food insecurity [31]. Price volatility is another pressing issues in Bangladesh, especially for the lower income groups who are forced to be dependent on cereal-based diets. Sometimes, seasonality also influences the dietary patterns in Bangladesh. Thus, diversification toward pulses, vegetables, and animal‑source foods requires supportive policies on pricing, and availability considering the affordability of the poorer community. Household decision making, food environment, and cultural preferences also influence the dietary choices of the households.”

Comment 12. The abstract should be shortened and focused on the main findings. Terminology should be standardized throughout. Vitamin B12 units in Table 3 should be corrected to µg, p- values should be reported as p < 0.001 rather than 0.000, and language editing is recommended.

Response: We appreciate the reviewer’s suggestions regarding abstract length, terminology, and editorial consistency. The number of words in the abstract has been kept within the journal’s limit and the main objectives and key findings are presented. Terminology has been standardized throughout the manuscript for consistency. In Table 3, Vitamin B12 units have been corrected to µg, and p‑values are now reported as p < 0.001 rather than 0.000. We have also undertaken language editing to enhance readability and precision.

Attachment Submitted filename: Authors response to reviewers comments.docx

# Peer review: Decision Letter 1

**DOI:** 10.1371/journal.pone.0359205.r003

**Authors:** Zehra Batu

10 Sep 2026

<p>Food Consumption Patterns and Nutrient Adequacy across Lifecycle Stages in Bangladesh

PONE-D-26-21024R1

Dear Dr. Md. Ruhul Amin

We’re pleased to inform you that your manuscript has been judged scientifically suitable for publication and will be formally accepted for publication once it meets all outstanding technical requirements.

Within one week, you’ll receive an e-mail detailing the required amendments. When these have been addressed, you’ll receive a formal acceptance letter and your manuscript will be scheduled for publication.

An invoice will be generated when your article is formally accepted. Please note, if your institution has a publishing partnership with PLOS and your article meets the relevant criteria, all or part of your publication costs will be covered. Please make sure your user information is up-to-date by logging into Editorial Manager at Editorial Manager® and clicking the ‘Update My Information' link at the top of the page. For questions related to billing, please contact billing support.

If your institution or institutions have a press office, please notify them about your upcoming paper to help maximize its impact. If they’ll be preparing press materials, please inform our press team as soon as possible -- no later than 48 hours after receiving the formal acceptance. Your manuscript will remain under strict press embargo until 2 pm Eastern Time on the date of publication. For more information, please contact onepress@plos.org.

Kind regards,

Zehra Batu

Academic Editor

PLOS One

Additional Editor Comments (optional):

Reviewers' comments:

Reviewer's Responses to Questions

**Comments to the Author**

Reviewer #2: All comments have been addressed

**********

2. Is the manuscript technically sound, and do the data support the conclusions?

Reviewer #2: Yes

**********

3. Has the statistical analysis been performed appropriately and rigorously?

Reviewer #2: Yes

**********

4. Have the authors made all data underlying the findings in their manuscript fully available?

The PLOS Data policy

Reviewer #2: Yes

**********

5. Is the manuscript presented in an intelligible fashion and written in standard English?

Reviewer #2: Yes

**********

Reviewer #2: Reviewer 2 comments on Revised version of the manuscript

I observed some key improvements:

1. Methodological Rigor: The authors clearly justified using both NSB and BIHS datasets, detailed how usual intake was calculated using variance decomposition, and explicitly stated the limitations of using the Adult Male Equivalent (AME) method and Indian EAR values.

2. Nutritional Depth: The discussion was substantially expanded to cover carbohydrate quality (polished rice vs. fiber/whole grains), antinutritional factors (phytates reducing iron and zinc bioavailability), hidden hunger in women of reproductive age, and the impact of urban dietary transitions.

3. Statistical & Visual Transparency: Model assumptions for the multivariate logistic regression (VIF, variable selection, MPA > 0.5 threshold) were fully specified, and Table 3 and p-value formatting issues were corrected.

Recommendation: Accept. Every critique was directly integrated into both the manuscript text and the limitations section, resulting in a much stronger, scientifically sound paper ready for publication.

**********

what does this mean?). If published, this will include your full peer review and any attached files.

If you choose “no”, your identity will remain anonymous but your review may still be made public.

**Do you want your identity to be public for this peer review?**  For information about this choice, including consent withdrawal, please see our Privacy Policy

Reviewer #2: No

**********

Attachment Submitted filename: Plos one -Nutrition_Bdesh-Review of revision-final.pdf

# Editor report: Acceptance letter

**DOI:** 10.1371/journal.pone.0359205.r004

**Authors:** Zehra Batu

PONE-D-26-21024R1

PLOS One

Dear Dr. Amin,

I'm pleased to inform you that your manuscript has been deemed suitable for publication in PLOS One. Congratulations! Your manuscript is now being handed over to our production team.

At this stage, our production department will prepare your paper for publication. This includes ensuring the following:

* All references, tables, and figures are properly cited

* All relevant supporting information is included in the manuscript submission,

* There are no issues that prevent the paper from being properly typeset

You will receive further instructions from the production team, including instructions on how to review your proof when it is ready. Please keep in mind that we are working through a large volume of accepted articles, so please give us a few days to review your paper and let you know the next and final steps.

Lastly, if your institution or institutions have a press office, please let them know about your upcoming paper now to help maximize its impact. If they'll be preparing press materials, please inform our press team within the next 48 hours. Your manuscript will remain under strict press embargo until 2 pm Eastern Time on the date of publication. For more information, please contact onepress@plos.org.

You will receive an invoice from PLOS for your publication fee after your manuscript has reached the completed accept phase. If you receive an email requesting payment before acceptance or for any other service, this may be a phishing scheme. Learn how to identify phishing emails and protect your accounts at https://explore.plos.org/phishing.

If we can help with anything else, please email us at customercare@plos.org.

Thank you for submitting your work to PLOS One and supporting open access.

Kind regards,

PLOS One Editorial Office Staff

on behalf of

Professor Zehra Batu

Academic Editor

PLOS One
